# Naturally acquired malaria is associated with shorter leukocyte relative telomere length and increased hTERT expression in an endemic nigerian population

**DOI:** 10.3389/fcimb.2026.1880313

**Published:** 2026-08-06

**Authors:** Theophilus Nang Wakai, Shalom Nwodo Chinedu, Israel Sunmola Afolabi

**Affiliations:** 1 Covenant Applied Informatics and Communication–Africa Centre of Excellence (CApIC-ACE), Ota, Nigeria; 2 Department of Biochemistry, College of Science and Technology, Covenant University, Ota, Nigeria; 3 Covenant University Public Health and Wellbeing Research Cluster (CUPHWERC), Covenant University, Ota, Nigeria

**Keywords:** host–pathogen interaction, immune-cell ageing, leukocyte telomere length, malaria, parasite density, telomerase reverse transcriptase (hTERT)

## Abstract

**Background:**

Population-level studies have linked malaria endemicity to shorter leukocyte telomere length, but the patient-level molecular basis of this association remains unknown. This study investigated the associations of microscopy-confirmed malaria with relative leukocyte telomere length (rTL), human telomerase reverse transcriptase (hTERT) expression, parasite density, and haematological parameters in an endemic Nigerian cohort.

**Methods:**

A total of 304 participants with complete microscopy and haematological data were included, comprising equal numbers of malaria-positive (n = 152) and malaria-negative (n = 152) individuals. From this cohort, an age-matched molecular subset of 40 participants (22 malaria-positive and 18 malaria-negative) was selected for rTL and hTERT analyses. Relative leukocyte telomere length was determined as the telomere-to-single-copy gene (T/S) ratio using 36B4, while hTERT expression was quantified by RT-qPCR using GAPDH as the housekeeping gene.

**Results:**

Malaria-positive participants had significantly lower haemoglobin and platelet counts than controls (both *p* < 0.001). In the molecular subset, malaria-positive participants had significantly shorter rTL than controls (*p* < 0.001), whereas hTERT expression was significantly higher (*p* = 0.004). Among malaria-positive participants, rTL differed across parasite-density categories (*p* = 0.011) and was inversely associated with parasite density (r = −0.68, *p* < 0.001). Exploratory multivariable analyses showed that malaria positivity and higher parasite density were independently associated with shorter relative leukocyte telomere length.

**Discussion/conclusion:**

To the best of our knowledge, this study is the first to provide patient-level evidence supporting the hypothesis that active malaria may contribute to the shorter relative leukocyte telomere length observed in malaria-endemic African populations. Longitudinal studies are needed to determine whether these changes are transient, cumulative, or reversible following treatment.

## 1 Introduction

Long before the 21st century, malaria had already been recognized as a major public health burden; despite sustained control efforts, it remains one of the most persistent infectious disease challenges, particularly in sub-Saharan Africa (Alharbi and Ahmed, 2026; Oladipo et al., 2022; World Health Organization, 2025). Conventional malaria diagnosis depends on parasite detection, yet parasite presence alone does not explain why patients with apparently comparable infections can show very different degrees of anaemia, thrombocytopenia, fever, and broader physiological disturbance (Li et al., 2025; Liu and Li, 2025; Buthelezi et al., 2025). This heterogeneity has intensified interest in host-response biology as a complementary lens for understanding malaria pathogenesis and recovery (Mukhiya et al., 2024; Wakai et al., 2026a).

At the cellular level, malaria imposes repeated oxidative and inflammatory insults (Vasquez et al., 2021; Bediako et al., 2019; Popa and Popa, 2021). Blood-stage parasite replication leads to erythrocyte rupture, haem release, inflammatory signaling, leukocyte activation, and increased proliferative demand (Burns et al., 2019; Bucşan and Williamson, 2020; Ourives et al., 2018). These same processes are mechanistically relevant to biological ageing because telomeric DNA is particularly vulnerable to oxidative damage and replication-associated (Singh et al., 2018; Armstrong and Boonekamp, 2023; Singh et al., 2019). Leukocyte telomere length has therefore emerged as an integrative marker of cumulative cellular burden in infection, inflammation, and chronic immune activation (Wakai et al., 2025b; Miglar et al., 2026).

The existing malaria-telomere literature is suggestive but incomplete. In avian malaria, chronic infection accelerated telomere degradation and senescence, and later work demonstrated parallel telomere shortening across multiple tissues (Asghar et al., 2016, 2015). In humans, acute malaria in travelers was associated with transient telomere shortening, reduced telomerase activity, and increased expression of senescence-related markers during early follow-up (Asghar et al., 2018), while controlled human malaria infection studies have also shown malaria-associated perturbation of cellular ageing biomarkers (Miglar et al., 2021). At the population level, a recent African analysis linked higher malaria endemicity with shorter leukocyte telomeres, but such ecological evidence does not directly demonstrate patient-level telomere shortening in microscopy-confirmed infection (McQuillan et al., 2024; Band and Leffler, 2024). Evidence from endemic human populations remains limited and somewhat context dependent. A recent longitudinal study in Kenyan children under active malaria surveillance found no evidence that cumulative symptomatic or asymptomatic *P. falciparum* exposure was associated with sustained telomere shortening; rather, telomere length shortens mainly with age, while asymptomatic parasitaemia at sampling showed only a modest, marginal positive association with telomere length (Miglar et al., 2026). This suggests that the effect of malaria on relatively shorter TLs may differ according to age, immune history, infection status, and timing of sampling.

A further unresolved question concerns telomerase regulation during malaria. Telomerase is a ribonucleoprotein enzyme complex that maintains telomere length by adding telomeric repeat sequences to chromosome ends. Its catalytic subunit, human telomerase reverse transcriptase (hTERT), provides the reverse-transcriptase activity required for telomere elongation, while the telomerase RNA component serves as the template for repeat synthesis (Razgonova et al., 2020; Wang et al., 2019). In most somatic cells, telomerase activity is low or absent, but it can be induced in highly proliferative compartments, including haematopoietic progenitors and activated immune cells such as T-lymphocytes, B-lymphocytes, and, under some conditions, monocyte/macrophage-lineage cells (Basu et al., 2025; Ellis et al., 2022; Wakai et al., 2026b).

hTERT expression may be induced by signals that drive immune-cell activation and proliferation, such as antigen stimulation, cytokine exposure, oxidative stress, DNA-damage responses, and the need to preserve replicative capacity during clonal expansion (Fu et al., 2025; Huang et al., 2024). This response is biologically expected, as threats to telomere integrity can activate compensatory telomere-maintenance mechanisms. During infection, antigen-driven lymphocyte expansion and inflammatory signalling may therefore stimulate hTERT expression as part of an adaptive response to increased cellular turnover and telomere stress (Rembiałkowska et al., 2025; Yik et al., 2021; Cao et al., 2019).

The present study examines whether malaria endemicity is linked to short rTLs in sub-Saharan African populations, using patient-level data from an endemic Nigerian cohort. We evaluated microscopy-confirmed malaria in relation to leukocyte telomere length, hTERT expression, parasite density, and haematological indices to determine how active infection relates to ageing biology. Specifically, we tested whether malaria positivity is associated with relative shorter telomeres, whether hTERT expression changes alongside shorter relative telomere length, and whether increasing parasite burden reflects the degree of this disruption. These findings provide direct clinical insight into how malaria infection may influence host telomere biology in a high-transmission setting.

## 2 Materials and methods

### 2.1 Study design and setting

This cross-sectional study was conducted between March and December 2025 at Covenant University Medical Centre (CUMC), located in Canaanland, Ota, Ogun State, Nigeria. The centre serves students, staff, church members, and residents of surrounding communities within a semi-urban malaria-endemic setting. The study area lies at approximately 6.7°N latitude and 3.2°E longitude and experiences a tropical climate with distinct wet and dry seasons, with malaria transmission occurring year-round and peaking during the rainy season. A prior cross-sectional study conducted within the same population assessed knowledge, attitudes, and practices (KAP) related to malaria prevention and control and reported gaps between awareness and effective preventive behaviour (Wakai et al., 2025a). The setting has also been described as having a symptomatic malaria prevalence of approximately 44% (Oranusi et al., 2025).

### 2.2 Study population and analytical cohorts

**Figure 1:**
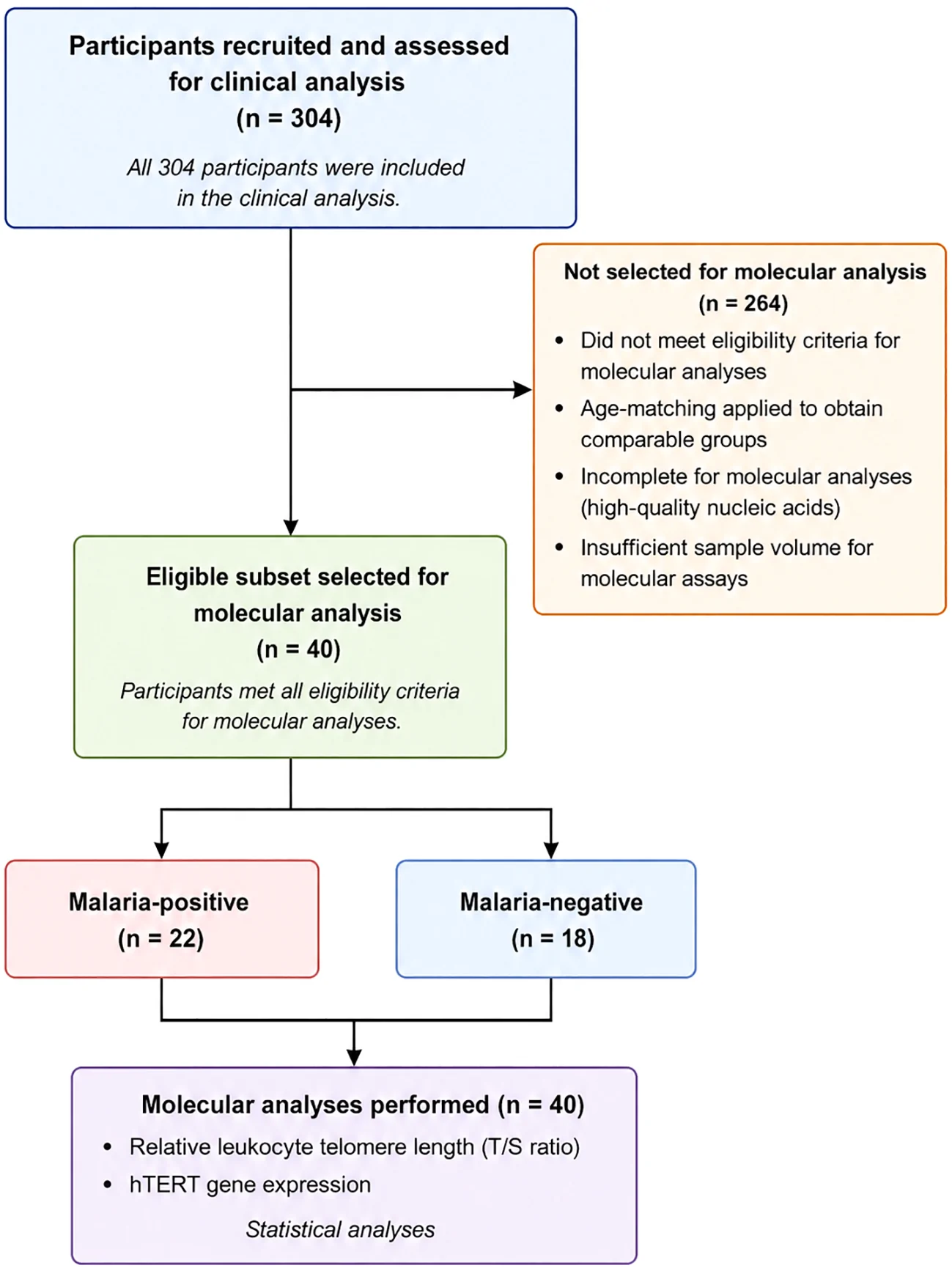
Flowchart of participant recruitment, classification, and selection for molecular analyses.

Participants were recruited through clinical presentations at the medical centre and community outreach screening activities. A total of 304 individuals with confirmed malaria status and complete clinical data were included, with equal representation of malaria-positive (n = 152) and malaria-negative (n = 152) participants. Participants were age-matched, and individuals with complete haematological data and available stored samples with adequate nucleic acid quality were included in a molecular subset (n = 40), comprising 22 malaria-positive and 18 malaria-negative participants (**Figure 1**). This subset was used to measure relative telomere length and hTERT expression and to assess their relationship with parasite density and haematological parameters.

### 2.3 Ethical considerations

Ethical approval for this study was obtained from the Covenant University Health Research Ethics Committee (CHREC/432/2024). Written informed consent was obtained from all adult participants, while assent was obtained from minors alongside consent from their parents or legal guardians where applicable. Administrative approval to conduct the study was obtained from the Director of Covenant University Medical Centre (CUMC) and relevant community leaders. All procedures involving human participants were conducted in accordance with the ethical standards of the institutional research committee and with the principles of the Declaration of Helsinki (World Medical Association, 2013).

### 2.4 Inclusion and exclusion criteria

Individuals presented for malaria screening or routine clinical evaluation at Covenant University Medical Centre, as well as those reached through community outreach activities, were eligible for inclusion. Participants were required to have resided in Canaanland for at least one year and to provide informed consent (or assent with parental/guardian consent for minors). Only individuals with complete parasitological and haematological data were included in the analytical cohort.

Participants were excluded if they were currently receiving antimalarial treatment or had taken antimalarial medication within the preceding three weeks. Individuals with incomplete clinical data, indeterminate malaria status, or inadequate sample quality were also excluded. For molecular analyses, only eligible participants with available stored samples that yielded amplifiable nucleic acid with acceptable purity (A260/A280 ratio within standard ranges) and consistent qPCR amplification were included (Lucena-Aguilar et al., 2016).

### 2.5 Sample collection and processing

Approximately 5 mL of venous blood was collected aseptically into EDTA-anticoagulated tubes for microscopy and haematological analysis. The buffy coat fraction was retained for molecular analyses. Samples designated for DNA and RNA extraction were stabilised using DNA/RNA Shield (Zymo Research, USA) and maintained under ultra-low temperature conditions until nucleic acid extraction. Extracted DNA and RNA were stored at −80 °C until downstream qPCR analysis.

### 2.6 Malaria diagnosis and parasite density determination

Malaria screening was initially performed using the CareStart Malaria HRP2/pLDH (Pf/PAN) Combo rapid diagnostic test (Access Bio, Somerset, NJ, USA), but microscopy-defined malaria status was used for the principal analytical comparisons. Thick and thin blood films were prepared on clean slides, stained with 10% Giemsa, and examined under oil immersion by trained microscopists. Thick films were used for parasite detection and quantification, whereas thin films supported morphological species identification.

Under oil immersion microscopy, asexual malaria parasites were counted against 200 white blood cells. Parasite density was expressed as parasites per microlitre of blood using an assumed standard leukocyte count of 8,000 WBCs/µL. Parasite density was determined from Giemsa-stained thick blood smears using standard malaria microscopy procedures. Parasite density was then expressed as parasites per microlitre of blood using the following formula:


$$\text{Parasite density }(\text{parasites}/\mu L)=\frac{\text{Number of asexual parasites counted}\times 8,000}{Number\;of\;WBCs\;counted}$$

Based on parasite density, malaria infections were categorised as low parasitaemia (<1,000 parasites/µL), moderate parasitaemia (1,000–9,999 parasites/µL), high parasitaemia (10,000–99,999 parasites/µL), and hyperparasitaemia (≥100,000 parasites/µL) (Awosolu et al., 2021; Wilairatana et al., 2013).

### 2.7 Haematological measurements and anaemia classification

Full blood count indices were obtained using Sysmex XS-1000i automated haematology analyser (Sysmex Corporation, Kobe, Japan). Haemoglobin concentration, haematocrit, red blood cell indices, total white blood cell count, differential counts, and platelet count were retained for analysis. Anaemia was defined and graded according to World Health Organization haemoglobin thresholds adjusted for age and sex. Participants were classified as non-anaemic, mildly anaemic, moderately anaemic, or severely anaemic based on the applicable WHO cut-off values as reported in related studies (Eyong et al., 2025; Wakai et al., 2026a).

Haemoglobin concentration was measured and recorded in grams per decilitre (g/dL). Haemoglobin concentration was recorded in grams per decilitre (g/dL), and anaemia severity categories were applied separately for children, adolescents, adult males, and adult females, as appropriate (Cheesbrough, 2006; Domenica Cappellini and Motta, 2015).

### 2.8 Relative telomere length determination

Relative leukocyte telomere length was determined by quantitative real-time PCR as the telomere-to-single-copy gene (T/S) ratio using the method described by Cawthon (2002) and Asghar et al. (2018). Genomic DNA was extracted from buffy coat fractions using the Quick-DNA™ Miniprep Plus Kit (D4068; Zymo Research, USA) according to the manufacturer's instructions. Samples designated for molecular analysis had been stabilized in DNA/RNA Shield (Zymo Research, USA) and stored under ultra-low temperature conditions until nucleic acid extraction. DNA quality and concentration were assessed prior to qPCR, and purified DNA was stored at −80 °C until analysis. The single-copy reference gene 36B4 (RPLP0) was used for telomere length quantification, while GAPDH was used exclusively as the housekeeping gene for hTERT RT-qPCR expression analysis. (**Section 2.9**).

Quantitative PCR reactions were performed on a QuantStudio™ 5 Real-Time PCR System (Applied Biosystems, USA) using Luna^®^ Universal qPCR Master Mix (M3003S; New England Biolabs, USA). Each 20 µL reaction contained 10 µL of 2× Luna Universal qPCR Master Mix, forward and reverse primers at 0.2–0.4 µM each, 1–2 µL of genomic DNA template, and nuclease-free water to the final volume. All reactions were performed in duplicate, and no-template controls were included in each run to monitor contamination.

The optimised qPCR cycling conditions consisted of an initial denaturation at 95 °C for 60 seconds, followed by 40 amplification cycles of denaturation at 95 °C for 15 seconds and annealing/extension at 60 °C for 30 seconds. Melt-curve analysis was performed after amplification to verify specificity. Mean cycle threshold (Ct) values from duplicate reactions were used for analysis.

### 2.9 Human telomerase reverse transcriptase expression analysis

Total RNA was extracted from buffy coat fractions using the Quick-RNA™ MiniPrep Plus Kit (R1057; Zymo Research, USA), according to the manufacturer’s instructions. Where required, DNase treatment was performed using DNase I, RNase-free (M0303S; New England Biolabs, USA). Extracted RNA was assessed for quality and concentration and stored at −80 °C until reverse transcription.

Complementary DNA (cDNA) was synthesised from total RNA using LunaScript^®^ RT SuperMix Kit (E3010L; New England Biolabs, USA), following the manufacturer’s protocol. The synthesised cDNA was used as the template for hTERT amplification. Expression of human telomerase reverse transcriptase (hTERT) was quantified by RT-qPCR using gene-specific primers, with GAPDH used as the housekeeping gene for normalisation.

RT-qPCR reactions were performed on a QuantStudio™ 5 Real-Time PCR System using Luna^®^ Universal qPCR Master Mix (M3003S; New England Biolabs, USA). Each 20 µL reaction contained 10 µL of 2× Luna Universal qPCR Master Mix, forward and reverse primers at 0.2–0.4 µM each, 1–2 µL of cDNA template, and nuclease-free water to the final volume. Reactions were performed in duplicate, and no-template controls were included in each run.

The thermal cycling conditions consisted of an initial denaturation at 95 °C for 60 seconds, followed by 40 amplification cycles of denaturation at 95 °C for 15 seconds and annealing/extension at 60 °C for 30 seconds. Melt-curve analysis was performed after amplification. Raw Ct values were averaged across duplicate reactions for both *hTERT* and *GAPDH*.

### 2.10 Primer validation and qPCR quality control

Gene-specific primers were designed using the PrimerQuest™ Tool (Integrated DNA Technologies, USA), based on target-gene mRNA sequences retrieved from the National Center for Biotechnology Information (NCBI) database. **Supplementary Table 1** provides the primer sequences, expected amplicon sizes, and assay-specific validation details. Primer performance was considered acceptable when amplification was specific, no-template controls showed no amplification, and replicate Ct values were consistent across duplicate reactions. Primer specificity was first evaluated in silico using NCBI Primer-BLAST and subsequently confirmed experimentally by agarose gel electrophoresis. PCR amplification products were separated on 1.5–2% agarose gels prepared in Tris–EDTA or 1× TBE buffer and visualised under UV illumination using a gel documentation system. The presence of bands within the expected amplicon size range, together with absence of amplification in no-template controls, confirmed primer specificity and suitability for qPCR analysis. All reactions were performed in duplicate, and mean Ct values were used for downstream analysis. Runs were accepted only when negative controls showed no amplification and replicate Ct values were consistent.

Intra-assay precision was assessed using duplicate reactions and expressed as the coefficient of variation (CV%). The mean intra-assay CVs were 0.46% for the telomere assay and 0.74% for the hTERT assay (**Supplementary Table 3**). All qPCR reactions were performed within a single analytical run; therefore, inter-assay precision was not evaluated because no independent runs were available to estimate between-run variability.

### 2.11 Statistical analysis

Continuous variables were assessed for distribution and are presented as medians with interquartile ranges [IQR]. Mann–Whitney U tests were used for two-group comparisons, while Kruskal–Wallis tests followed by Dunn’s *post hoc* tests were used for comparisons across parasite-density categories. Categorical variables are presented as frequencies and percentages and were compared using chi-square or Fisher’s exact tests, as appropriate. Spearman’s rank correlation assessed associations among parasite density, haematological indices, relative telomere length, and hTERT expression. Exploratory ordinary least squares regression models were used to estimate independent associations with rTL and hTERT expression. Regression results are reported as coefficients, 95% confidence intervals, and p-values. All tests were two-sided, with p < 0.05 considered statistically significant. Because of the modest molecular subset, multivariable models were considered exploratory and were used to evaluate the direction and robustness of associations rather than to generate definitive predictive estimates.

## 3 Results

### 3.1 Distribution of malaria positivity by age and sex

Age distribution did not differ significantly between malaria-positive and malaria-negative participants (p = 0.918). When participants were grouped into malaria-relevant age strata, microscopy prevalence was highest among participants aged 5–14 years and those older than 40 years, although this difference was not statistically significant (p = 0.325). Sex distribution was also not significantly associated with malaria positivity (p = 0.358) (**Table 1**).

**Table 1:** Microscopy-defined malaria positivity by age group and sex.

Variable	Category	Total n	Positive n	Negative n	Malaria positivity %	P-value
Age group	5–14 years	73	40	33	54.8	p = 0.325
	15–40 years	167	77	90	46.1	
	>40 years	64	35	29	54.7	
Sex	Female	159	84	75	52.8	p = 0.358
	Male	145	68	77	46.9	

### 3.2 Haematological profile by malaria status

Microscopy-positive participants showed the expected malaria-associated haematological disturbance. Compared with microscopy-negative controls, malaria-positive participants had lower red cell indices, haemoglobin, haematocrit and platelet counts, while temperature, WBC count and neutrophil percentage were higher. The strongest differences were observed for haemoglobin and platelet count, supporting anaemia and thrombocytopenia as core clinical features of malaria-associated physiological disturbance in this cohort (**Table 2**).

**Table 2:** Selected clinical and haematological variables by microscopy-defined malaria status.

Variable	Malaria-positive median [IQR]	Malaria-negative median [IQR]	p value
Age (years)	28 [14-40]	27 [17-38]	0.918
Temperature (°C)	38.59 [36.87-39.13]	36.74 [36.51-37.12]	<0.001
RBC (x10^12^/L)	4.07 [3.74-4.42]	5.04 [4.64-5.43]	<0.001
Haemoglobin (g/dL)	10.20 [8.30-11.71]	13.79 [12.55-14.88]	<0.001
Haematocrit (%)	33.12 [28.55-36.37]	42.05 [39.31-44.81]	<0.001
MCV (fL)	81.75 [77.11-89.35]	88.88 [84.79-94.06]	<0.001
MCH (pg)	27.93 [24.73-29.90]	30.08 [28.33-31.15]	<0.001
MCHC (g/dL)	32.18 [30.98-33.59]	33.99 [33.10-35.11]	<0.001
WBC (x10^9^/L)	7.85 [6.16-9.16]	6.44 [5.33-7.62]	<0.001
Neutrophils (%)	57.49 [52.24-64.01]	53.36 [47.63-58.68]	<0.001
Lymphocytes (%)	32.16 [26.65-38.70]	35.62 [31.66-39.92]	<0.001
Monocytes (%)	4.80 [3.44-6.52]	5.22 [3.33-6.52]	0.663
Eosinophils (%)	2.33 [1.44-3.14]	2.94 [2.10-3.99]	<0.001
Platelets (×10^9^/L)	142.94 [103.65-173.38]	260.48 [222.82-308.96]	<0.001

### 3.3 Anaemia distribution and parasite-density categories

Anaemia was more frequent and more severe among microscopy-positive participants than among microscopy-negative participants. Among malaria-positive participants, only 32/152 (21.1%) were non-anaemic, while 120/152 (78.9%) had some degree of anaemia. Moderate anaemia was the most common category among malaria-positive participants, affecting 68/152 (44.7%), followed by severe anaemia in 29/152 (19.1%) and mild anaemia in 23/152 (15.1%). In contrast, most malaria-negative participants were non-anaemic, with 133/152 (87.5%) having haemoglobin values within the non-anaemic range. Mild anaemia was observed on 18/152 (11.8%), while moderate anaemia was rare, occurring on only 1/152 (0.7%). No severe anaemia was observed among malaria-negative participants (**Table 2**).

Among microscopy-positive participants, parasite-density distribution showed a relatively even spread across categories. Low parasitaemia was observed in 36/152 (23.7%), moderate parasitaemia in 35/152 (23.0%), high parasitaemia in 41/152 (27.0%), and hyperparasitaemia in 40/152 (26.3%). This indicates that a substantial proportion of malaria-positive participants had high parasite burdens, with more than half classified as having either high parasitaemia or hyperparasitaemia (**Table 3**).

**Table 3:** Anaemia distribution and parasite-density categories in the analytical cohort.

Category	Malaria-positive n (%)	Malaria-negative n (%)
Anaemia distribution		
No anemia	32 (21.1)	133 (87.5)
Mild	23 (15.1)	18 (11.8)
Moderate	68 (44.7)	1 (0.7)
Severe	29 (19.1)	0 (0.0)
Parasite-density categories among microscopy-positive participant		
Low	36 (23.7)	–
Moderate	35 (23.0)	
High	41 (27.0)	
Hyperparasitaemia	40 (26.3)	

Anaemia percentages were calculated within each microscopy-status group. Parasite-density percentages were calculated among malaria-positive participants only.

### 3.4 Relative telomere length and hTERT expression by malaria status

**Figure 2:**
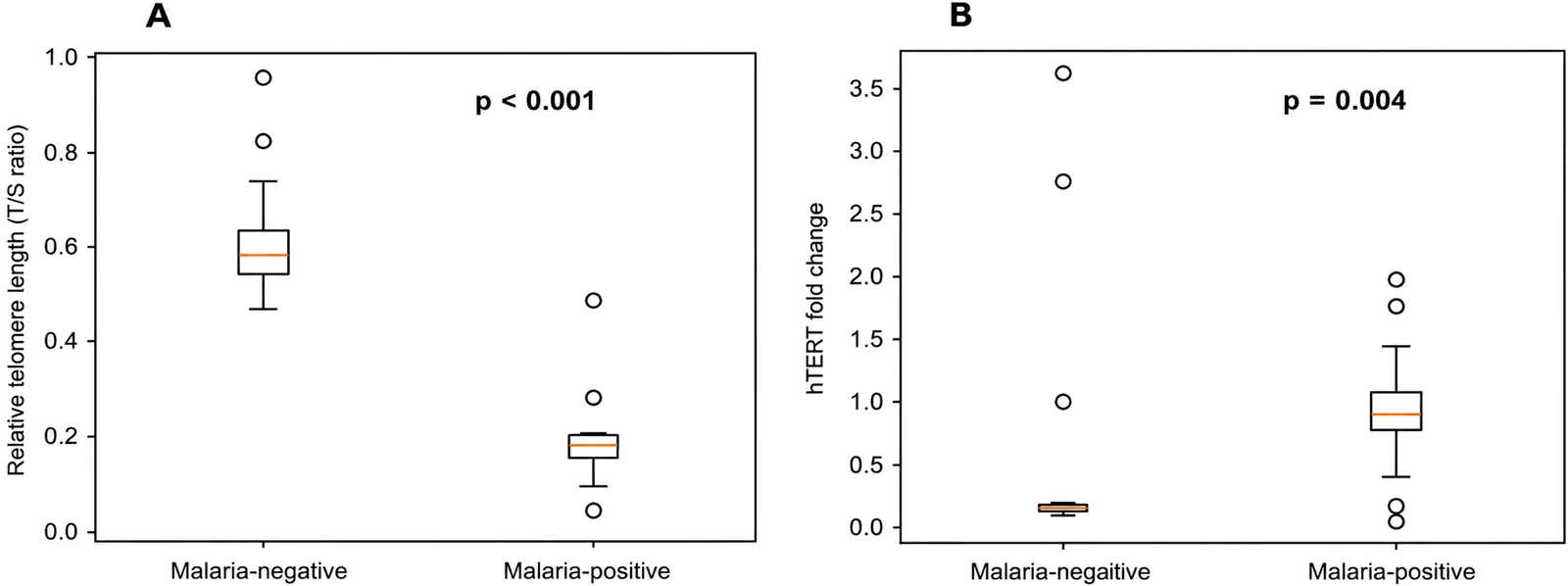
Relative telomere length **(A)** and hTERT expression **(B)** by malaria status. Boxplots showing rTL, expressed as the telomere-to-single-copy gene ratio (T/S ratio), and human telomerase reverse transcriptase (hTERT) fold change among microscopy-positive and microscopy-negative participants. Malaria-positive participants showed significantly reduced rTL and increased hTERT expression compared with malaria-negative participants.

In the molecular subset, the Median relative telomere length, expressed as the T/S ratio, was markedly lower in malaria-positive participants than in malaria-negative participants: 0.199 [0.172–0.216] versus 0.584 [0.554–0.636], respectively (p < 0.001; Cliff’s δ = −0.98) (**Figure 2A**). In contrast, hTERT expression was significantly higher in malaria-positive participants than in malaria-negative participants: 0.870 [0.770–1.050] versus 0.145 [0.133–0.170], respectively (p = 0.004; Cliff’s δ = 0.53) (**Figure 2B**).

### 3.5 Telomere and hTERT expression patterns across parasite-density categories

**Figure 3:**
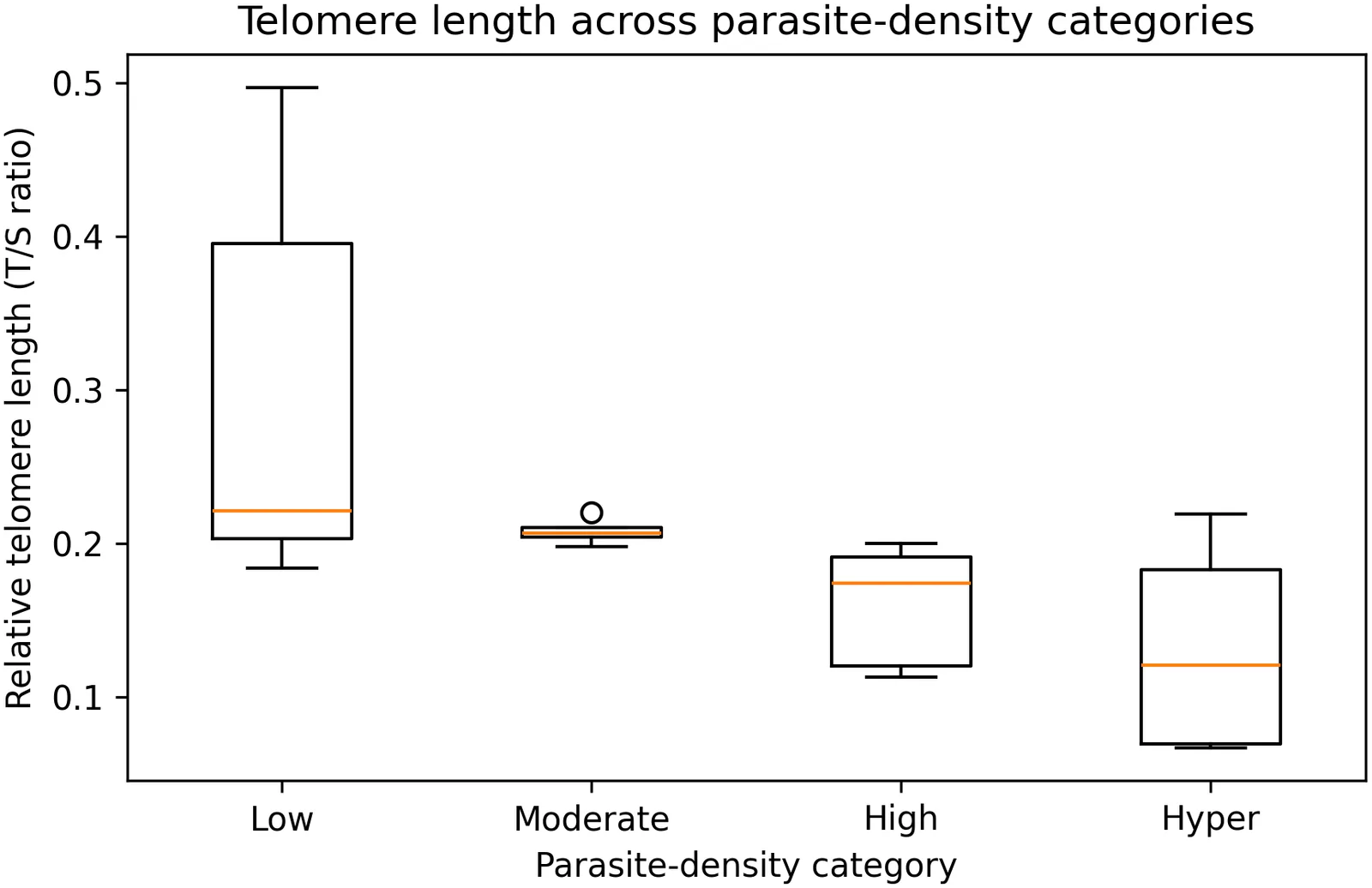
Relative telomere length across parasite-density categories among malaria-positive participants.

**Figure 4:**
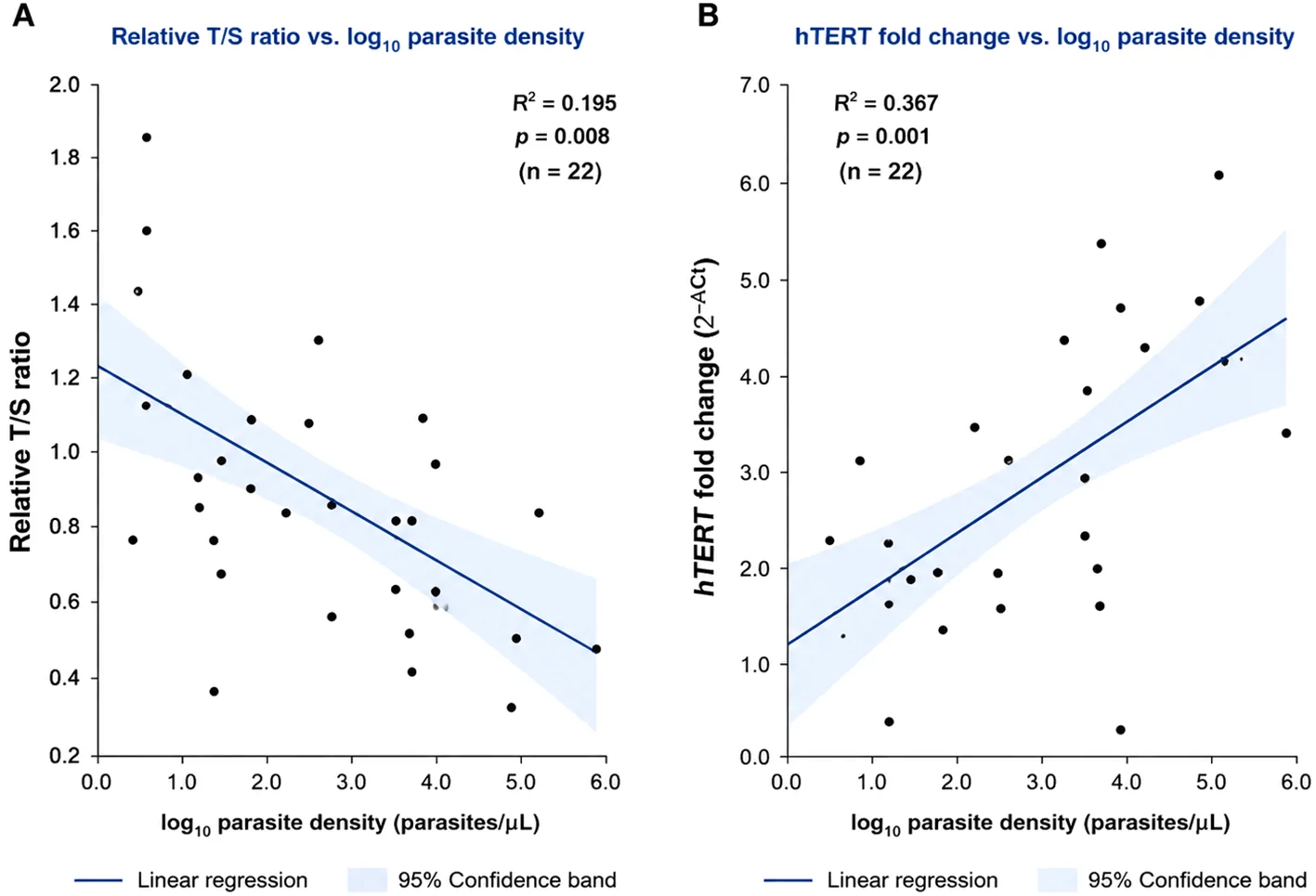
Scatter plots showing the relationships between **(A)** relative leukocyte telomere length (T/S ratio) and log_10_ parasite density, and **(B)** relative hTERT expression (2^−ΔCt^) and log_10_ parasite density among malaria-positive participants (n = 22). The solid blue lines represent the fitted linear regression models, and the shaded areas indicate the 95% confidence intervals. Corresponding R² and p values are displayed within each panel.

Within malaria-positive participants, relative telomere length showed a relatively shorter progress across parasite-density categories, with the lowest median T/S ratio observed among participants with hyperparasitaemia (**Figure 3**). Median relative telomere length decreased from 0.221 [0.203–0.395] in the low-parasitaemia group to 0.121 [0.069–0.183] in the hyperparasitaemia group, and this difference was statistically significant (p = 0.011). In contrast, hTERT expression did not differ significantly across parasite-density categories (p = 0.426), although the highest median hTERT fold change was observed among participants with hyperparasitaemia. Haemoglobin and platelet count also varied across parasite-density categories, but these differences were not statistically significant (**Table 4**). To further examine the continuous relationships between parasite density, relative leukocyte telomere length, and hTERT expression, scatter plots with fitted linear regression lines and 95% confidence bands were generated for malaria-positive participants (**Figure 4**).

**Table 4:** Relative telomere length, hTERT expression and selected haematological variables across parasite-density categories among malaria-positive participants.

Variable	Low	Moderate	High	Hyperparasitaemia	p value
rTL (T/S ratio)	0.221 [0.203-0.395]	0.206 [0.204-0.210]	0.174 [0.120-0.191]	0.121 [0.069-0.183]	0.011*
hTERT fold change	0.830 [0.500-0.860]	0.900 [0.640-0.968]	0.890 [0.770-1.330]	1.370 [1.085-1.505]	0.426
Haemoglobin (g/dL)	11.49 [10.98-12.05]	12.02 [11.05-12.80]	8.96 [7.55-11.32]	9.13 [7.51-10.88]	0.168
Platelets (×10^9^/L)	143.87 [95.02-167.08]	124.83 [100.23-146.43]	181.01 [142.00-186.35]	131.24 [108.18-151.56]	0.375

### 3.6 Relative telomere length across age and malaria status

**Figure 5:**
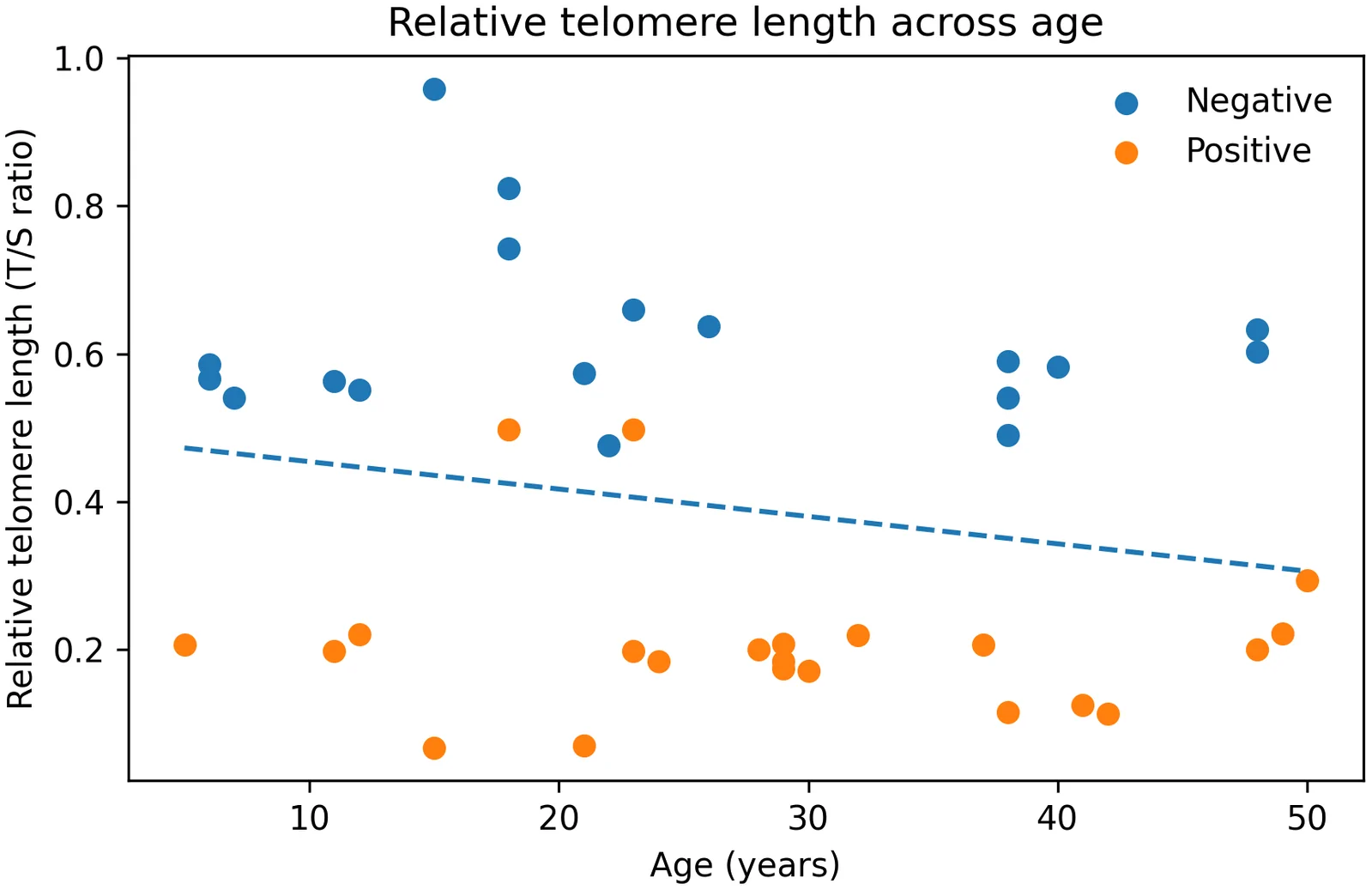
Relative telomere length across age by malaria status.

Relative telomere length was plotted against age by malaria status (**Figure 5**). Across the molecular subset, relative telomere length showed a weak inverse association with age, which was not statistically significant (Spearman ρ = −0.171; p = 0.292). Malaria-positive participants were predominantly clustered at lower T/S ratio values across the age range, whereas malaria-negative participants were generally clustered at higher T/S ratio values.

### 3.7 Correlation and multivariable analysis

To evaluate whether the reduction in relative telomere length was associated with malaria status independent of age, exploratory multivariable regression was performed using relative telomere length as the outcome variable, with malaria status, age, and sex included as predictors. Correlation and exploratory multivariable analyses were performed to evaluate the relationships among parasite density, haematological indices, relative telomere length, and human telomerase reverse transcriptase (hTERT) expression.

Among microscopy-positive participants, parasite density showed a strong inverse correlation with relative telomere length (ρ = −0.682; p < 0.001; **Table 5**). Relative telomere length showed a positive but non-significant correlation with haemoglobin concentration (ρ = 0.392; p = 0.071). No significant correlations were observed between relative telomere length and platelet count, temperature, or age. hTERT expression showed a positive but non-significant correlation with parasite density among microscopy-positive participants (ρ = 0.381; p = 0.080; **Table 5**). *hTERT* expression was not significantly correlated with haemoglobin concentration, platelet count, temperature, or age within the malaria-positive group.

**Table 5:** Spearman correlations among malaria-positive participants.

Marker	Clinical variable	N	Spearman rho	p value
T/S ratio	Parasite density	22	-0.682	<0.001***
	Haemoglobin	22	0.392	0.071
	Platelets	22	-0.262	0.239
	Temperature	22	-0.146	0.515
	Age	22	-0.002	0.992
hTERT fold change	Parasite density	22	0.381	0.080
	Haemoglobin	22	-0.128	0.569
	Platelets	22	-0.095	0.674
	Temperature	22	0.313	0.156
	Age	22	-0.042	0.853

Exploratory multivariable regression was performed to assess whether malaria status remained associated with relative telomere length after adjustment for demographic, haematological, and leukocyte differential variables. In the full molecular subset, malaria positivity remained independently associated with lower relative telomere length after adjustment for age, sex, haemoglobin concentration, platelet count, neutrophil percentage, and lymphocyte percentage (β = −0.336; 95% CI: −0.483 to −0.189; *p* < 0.001; R² = 0.824; **Table 6**). Male sex showed a positive but non-significant association with relative telomere length (β = 0.072; 95% CI: −0.003 to 0.146; *p* = 0.058). Age, haemoglobin concentration, platelet count, neutrophil percentage, and lymphocyte percentage were not significant predictors of relative telomere length. Specifically, neither neutrophil percentage (β = −0.002; 95% CI: −0.007 to 0.004; *p* = 0.563) nor lymphocyte percentage (β = 0.000; 95% CI: −0.006 to 0.006; *p* = 0.894) was independently associated with relative telomere length, indicating that the observed association between malaria and shorter relative leukocyte telomere length was not explained by variation in these major circulating leukocyte populations.

**Table 6:** Exploratory multivariable regression models for relative telomere length and hTERT expression.

Model	Predictor	Beta/coefficient (β)	SE	p value	95% CI low	95% CI high	R²
T/S model	Intercept	0.412	0.251	0.111	-0.100	0.923	0.824
	Malaria positivity	-0.336	0.072	<0.001***	-0.483	-0.189	0.824
	Age (years)	-0.001	0.001	0.465	-0.004	0.002	0.824
	Male sex	0.072	0.037	0.058	-0.003	0.146	0.824
	Haemoglobin (g/dL)	0.017	0.010	0.117	-0.004	0.038	0.824
	Platelets (×10^9^/L)	0.000	0.000	0.765	-0.001	0.001	0.824
	Neutrophils (%)	-0.002	0.003	0.563	-0.007	0.004	0.824
	Lymphocytes (%)	0.000	0.003	0.894	-0.006	0.006	0.824
T/S positive-only model	Intercept	0.503	0.218	0.035*	0.040	0.966	0.441
	log_10_ parasite density	-0.063	0.027	0.030*	-0.119	-0.007	0.441
	Age (years)	-0.001	0.002	0.514	-0.005	0.003	0.441
	Male sex	0.024	0.045	0.599	-0.072	0.120	0.441
	Haemoglobin (g/dL)	0.003	0.012	0.824	-0.022	0.028	0.441
	Platelets (×10^9^/L)	-0.000	0.001	0.486	-0.001	0.001	0.441
hTERT model	Intercept	-0.887	1.085	0.419	-3.092	1.317	0.300
	T/S ratio	3.709	1.137	0.003**	1.398	6.020	0.300
	Malaria positivity	1.785	0.519	0.002**	0.730	2.839	0.300
	Age (years)	-0.006	0.009	0.536	-0.024	0.013	0.300
	Male sex	-0.220	0.245	0.377	-0.718	0.279	0.300
	Haemoglobin (g/dL)	-0.047	0.068	0.493	-0.184	0.091	0.300

Within microscopy-positive participants, log_10_ parasite density remained independently associated with lower relative telomere length after adjustment for age, sex, haemoglobin concentration, and platelet count (β = −0.063; 95% CI: −0.119 to −0.007; *p* = 0.030; R² = 0.441; **Table 6**). Age, sex, haemoglobin concentration, and platelet count were not significant predictors in the positive-only model.

In the hTERT model, relative telomere length was positively associated with hTERT expression (β = 3.709; 95% CI: 1.398 to 6.020; *p* = 0.003; R² = 0.300; **Table 6**). Malaria positivity was also independently associated with higher hTERT expression (β = 1.785; 95% CI: 0.730 to 2.839; *p* = 0.002). Age, sex, and haemoglobin concentration were not significant predictors of hTERT expression.

## 4 Discussion

In this study, we examined leukocyte telomere biology in relation to microscopy-confirmed malaria, parasite density, hTERT expression, and routine haematological indices in an endemic Nigerian cohort. The principal finding was that malaria-positive participants had markedly shorter relative telomere length than malaria-negative participants, while hTERT expression was higher in the same infected group. The association with shorter relative leukocyte telomere length was not explained by age distribution: age was similar between malaria-positive and malaria-negative participants, showed only a weak non-significant correlation with relative telomere length in the molecular subset, and was not an independent predictor in the adjusted model. Instead, malaria positivity and parasite density showed the strongest molecular signals. Within infected participants, relative telomere length lowers across parasite-density categories and correlated inversely with parasite density, indicating an association between higher parasite density and shorter relative leukocyte telomere length.

These findings should be interpreted within the context of malaria pathobiology rather than as an isolated molecular observation. Malaria-positive participants showed the expected clinical and haematological features of malaria-associated physiological disturbance, including lower haemoglobin, haematocrit, red cell indices, and platelet counts compared with microscopy-negative participants (Awoke and Arota, 2019; Naser et al., 2024). Anaemia was concentrated in the malaria-positive group, and all severe anaemia cases occurred among infected participants. The telomere signal therefore occurred against a biologically coherent background of haemolysis, thrombocytopenia, fever, and leukocyte perturbation. This internal consistency strengthens the plausibility that the observed telomere changes reflect infection-associated host stress rather than a random difference between groups.

The present results are consistent with the broader concept that malaria can impose hidden cellular costs beyond immediate parasite detection. Experimental avian studies first showed that chronic malaria infection may accelerate shortening of leukocyte telomere length, with consequences for survival and fitness (Asghar et al., 2015). In humans, acute malaria in non-immune travellers was associated with transient leukocyte shorter relative telomere length and altered cellular ageing markers during follow-up (Asghar et al., 2018). Controlled human malaria infection studies similarly showed that malaria can perturb cellular ageing biomarkers in malaria-naive adults (Miglar et al., 2021). The present work adds a distinct clinical perspective by studying naturally acquired infection in an endemic African setting, where individuals may have prior exposure, partial immunity, and concurrent environmental pressures that differ substantially from traveller or challenge-study populations. (Wakai et al., 2025)

Our findings also complement recent population-level evidence linking malaria endemicity to shorter leukocyte telomeres in sub-Saharan African populations (McQuillan et al., 2024). Population-level endemicity studies are valuable because they capture broad exposure landscapes, but they cannot by themselves determine whether an actively infected patient has measurable telomere disruption at the point of clinical assessment. By showing shorter relative telomere length among malaria-positive participants and an inverse parasite-density gradient within infected individuals, this study provides patient-level evidence that active malaria and parasite burden are associated with relative leukocyte TLs in a real-world endemic setting.

At the same time, the findings should not be presented as evidence that all malaria exposure inevitably causes permanent shorter relative TL. Miglar et al. recently reported no evidence that cumulative symptomatic or asymptomatic *P. falciparum* exposure was associated with sustained shorter leukocyte telomere length in Kenyan children under long-term surveillance (Miglar et al., 2026). Their study addressed cumulative exposure over childhood, whereas the present study captured active malaria infection and parasite-density gradients at clinical presentations. These are related but not identical biological questions. Their telomere measurements were taken at three-year intervals, which may be better suited to detecting long-term trajectories than short-lived changes during acute inflammatory episodes. In contrast, our data suggests that the intensity of current infection may be associated with shorter TL at the time of presentation. Collectively, these findings support a more nuanced model in which malaria-associated telomere changes may depend on timing of sampling, clinical state, parasite burden, immune history, age structure, and the balance between inflammatory activation and immune regulation (Andreu-Sánchez et al., 2022; Fyhrquist and Saijonmaa, 2016).

The difference between symptomatic clinical infection and asymptomatic or cumulative exposure is particularly important. In endemic settings, repeated exposure can generate anti-parasite immunity and immunoregulatory responses that reduce inflammatory pathology despite persistent or recurrent parasitaemia (Boyle et al., 2024; de Mendonça and Barral-Netto, 2015). This may partly explain why cumulative exposure in children under surveillance may not produce the same telomere pattern observed during acute malaria in non-immune adults or during active clinical presentations. In our cohort, the parasite-density gradient was one of the clearest signals: participants with higher parasite burdens had shorter relative telomere length (**Figure 3**). This suggests that parasite burden at the time of sampling may be a more sensitive indicator of acute cellular stress than binary infection status alone, particularly when the aim is to capture host injury during active disease rather than long-term cumulative exposure.

Several mechanisms could plausibly link active malaria to shorter leukocyte telomeres. Blood-stage malaria causes repeated erythrocyte rupture, haemoglobin release, free haem exposure, inflammatory signalling, and oxidative stress (Mohandas and An, 2012; Vasquez et al., 2021; Nassour et al., 2024; Tunnicliffe et al., 2025). Telomeric DNA is guanine-rich and particularly vulnerable to oxidative damage; therefore, the inflammatory and pro-oxidant environment generated during malaria may accelerate telomere erosion in circulating leukocytes (Singh et al., 2019; Armstrong and Boonekamp, 2023; Voros et al., 2025). In parallel, antigen-driven immune activation may increase leukocyte proliferation and turnover (Paiardini and Müller-Trutwin, 2013; Heinzel et al., 2018; Moro-García et al., 2018). Increased proliferation can shorten telomeres through replication-associated sequence loss, while oxidative damage can further compromise telomere integrity (Wakai et al., 2025b). The observed inverse association between parasite density and relative telomere length is consistent with this combined oxidative and replicative stress model.

The higher hTERT expression observed among malaria-positive participants should be interpreted carefully. hTERT encodes the catalytic subunit of telomerase, but hTERT gene expression is not equivalent to direct telomerase enzyme activity, and neither is interchangeable with telomere length. In this study, higher hTERT expression occurred alongside shorter relative telomere length. This apparent discordance is biologically plausible. During infection, antigen stimulation, cytokine exposure, oxidative stress, and DNA damage responses can induce telomerase-related pathways in activated immune cells as part of a compensatory attempt to maintain replicative capacity (Cao et al., 2019; Yik et al., 2021; Huang et al., 2024). However, such activation may be insufficient to prevent telomere loss under intense or sustained inflammatory stress. Thus, the concurrent pattern of reduced telomere length and increased hTERT expression is more consistent with telomere-maintenance activation under stress than with successful telomere restoration (Gupta et al., 2026; Wakai et al., 2025b).

The hTERT findings also align with the idea that telomere length changes during infection are regulated by more than a simple linear telomerase-response mechanism. Miglar et al. emphasised that telomere length, shorter leukocyte telomere length rate, and telomerase activity are related but distinct biological measures (Miglar et al., 2026). In their controlled human malaria infection study, Miglar et al. (2021) reported telomerase activation after antimalarial treatment, supporting the possibility that telomerase-related responses may be part of post-infection recovery rather than a direct marker of preserved telomere length. This is also consistent with the longitudinal traveller study by Asghar et al. (2018), in which acute malaria was followed by dynamic telomere and telomerase changes over time. In the present study, the coexistence of shorter relative telomere length and higher hTERT expression may therefore represent a compensatory but incomplete telomere-maintenance response during active malaria infection. Here, we measured relative telomere length and hTERT expression, but not telomerase activity. Therefore, the data cannot determine whether increased hTERT transcription translated into active telomere elongation. The positive association between malaria positivity and hTERT expression nevertheless suggests that active infection was accompanied by transcriptional activation of a telomerase-related pathway (**Figure 2B**). Future studies should combine hTERT expression with direct telomerase activity assays, telomere damage markers, and immune-cell phenotyping to determine whether this response is protective, insufficient, or maladaptive.

Age is a central consideration in any telomere study because leukocyte telomere length generally shortens with increasing chronological age (Adegunsoye et al., 2023; Vaiserman and Krasnienkov, 2021). In the present work, however, age was not the main statistical signal. The age distribution was balanced between malaria-positive and malaria-negative participants in the broader analytical cohort, the correlation between age and relative telomere length was weak and not significant, and age did not independently predict relative telomere length in the adjusted model (**Figure 5**). This does not mean that age is biologically irrelevant; rather, within the age range and sample structure studied here, malaria status and parasite density showed stronger associations with relative telomere length than chronological age. This point is important because it reduces, but does not eliminate, concern that the observed telomere difference simply reflects age imbalance.

The haematological findings further support clinical relevance of the molecular results. Anaemia and thrombocytopenia are well-recognised features of malaria and reflect the combined effects of erythrocyte destruction, splenic clearance, inflammatory suppression of erythropoiesis, platelet activation, and immune-mediated peripheral destruction (Erhabor et al., 2014; Krishna and Chalamalasetty, 2023; Liu and Li, 2025; Eskandari et al., 2018; Achame et al., 2025). In this study, malaria-positive participants had substantially lower haemoglobin and platelet counts than malaria-negative participants, and anaemia severity was concentrated among infected individuals. Although haemoglobin and platelet count were not independent predictors of relative telomere length in all models, their directionality and distribution place the telomere signal within a broader host-injury phenotype. This supports the view that shorter leukocyte telomere length may be one component of a wider physiological response to malaria-associated haemolytic and inflammatory stress (Nassour et al., 2024; Udobi et al., 2026; Wakai et al., 2026a).

This study has several strengths. First, malaria status was defined by microscopy for the primary comparisons, and parasite density was quantified among infected participants, enabling both infection-status and parasite-burden analyses. Second, the analytical cohort included equal numbers of malaria-positive and malaria-negative participants with complete microscopy and haematological data, reducing imbalance in the main comparison. Third, molecular assays were performed on buffy coat–derived nucleic acids, supporting interpretation of telomere outcomes as leukocyte relative telomere length. Fourth, telomere and hTERT measurements were analysed alongside routine haematological indices, allowing the molecular findings to be interpreted in the context of established malaria-associated haematological disturbances. Finally, age-adjusted and covariate-adjusted exploratory models directly addressed a common concern in telomere studies: whether apparent infection effects reflect underlying age differences.

The limitations also need careful consideration. The cross-sectional design prevents inference about temporal sequence, persistence, reversibility, or causality. It is not possible to determine whether shorter relative leukocyte telomere length preceded infection, occurred during acute infection, or would recover after treatment. The molecular subset was modest in size, particularly for parasite-density subgroup comparisons; therefore, the density-gradient findings should be interpreted as strong preliminary evidence requiring validation. Telomere length was measured in bulk leukocyte-derived material rather than purified immune-cell subsets, so changes may reflect both true shorter TLs within cells and shifts in leukocyte composition. Because relative telomere length was measured in DNA extracted from whole blood, differences in leukocyte composition could potentially influence the results. To address this possibility, neutrophil and lymphocyte percentages were included as covariates in the multivariable regression model. Neither variable was independently associated with relative leukocyte telomere length, whereas the association between malaria status and shorter relative leukocyte telomere length remained statistically significant after adjustment.

These findings suggest that the observed relatively short TL reflects an association with malaria infection itself rather than being driven by shifts in major circulating leukocyte populations. Nevertheless, we acknowledge that complete immune-cell heterogeneity cannot be excluded because telomere length was not measured in purified leukocyte subsets. Future studies should combine telomere measurement with immune-cell phenotyping or cell-sorted analyses (Trigueros et al., 2026).

Undetected co-infections or recent inflammatory illnesses may also influence telomere biology and could not be fully excluded. hTERT was measured at the gene-expression level and not as telomerase enzyme activity. Other inflammatory, nutritional, genetic, and environmental factors that can influence telomere biology, including recent infections, micronutrient status, haemoglobinopathies, and chronic inflammatory conditions, were not fully characterized. Finally, because qPCR-based telomere measurement provides a relative estimate, the results should be interpreted as relative leukocyte telomere differences rather than absolute telomere length in kilobases. The multivariable regression models should also be interpreted cautiously because of the modest molecular subset and the number of covariates included. These models were exploratory and intended to assess whether the observed associations remained directionally consistent after adjustment, rather than to provide definitive prediction estimates.

These limitations do not negate the main finding, but they define its proper interpretation. The data support an association between active naturally acquired malaria, parasite burden, shorter leukocyte relative telomere length, and increased hTERT expression in this Nigerian cohort. They do not prove permanent telomere damage or establish that malaria alone is responsible for all observed telomere variation. The most defensible interpretation is that active malaria was associated with a cellular stress profile in circulating leukocytes, characterised by shorter relative leukocyte telomere length and transcriptional activation of a telomerase-related pathway. This profile was most evident among participants with higher parasite density and occurred alongside malaria-associated anaemia and thrombocytopenia.

Overall, the multivariable analyses demonstrated a consistent pattern of associations, with malaria infection remaining independently associated with relative leukocyte telomere length and hTERT expression after adjustment for potential confounders. The lack of independent effects from age, sex, and routine haematological indices suggests that these host characteristics do not substantially explain variability in telomere length or hTERT expression within the observed context (**Table 6**). Instead, the findings are consistent with malaria being associated with an inflammatory and proliferative stress profile linked to alterations in cellular ageing pathways. The positive relationship between telomere length and hTERT expression further suggests a compensatory telomerase response to infection-associated shorter relative leukocyte telomere length, potentially reflecting an attempt to preserve cellular replicative capacity under sustained immune activation (Miglar et al., 2021). Collectively, these findings suggest that malaria-related biological stress is linked to shorter relative leukocyte telomere length and increased hTERT expression, whereas baseline demographic characteristics and routine haematological parameters showed no independent associations with these molecular outcomes.

## 5 Conclusion

This study found that naturally acquired clinical malaria was associated with shorter relative leukocyte telomere length and higher hTERT expression in an endemic Nigerian population. The inverse association between parasite density and relative leukocyte telomere length further suggests that parasite burden may be associated with variation in host telomere biology during active infection. These findings complement previous observations from experimental, clinical, and population-based studies by providing patient-level molecular data from individuals with microscopy-confirmed malaria in a malaria-endemic African setting. To the best of our knowledge, the findings are consistent with the hypothesis that active malaria may contribute to the shorter leukocyte telomere length observed in malaria-endemic populations. However, the cross-sectional design precludes conclusions regarding causality or the temporal sequence of these associations. Longitudinal studies incorporating pre-infection, acute infection, post-treatment, and recovery sampling, together with cell-specific telomere analyses, direct telomerase activity measurements, inflammatory and oxidative stress biomarkers, and host genetic factors, are required to determine whether the observed telomere changes are transient, cumulative, reversible, or clinically prognostic.

## Data Availability

The original contributions presented in the study are included in the article/**Supplementary Material**. Further inquiries can be directed to the corresponding author.
