# Supplementary material for: Naturally acquired malaria is associated with shorter leukocyte relative telomere length and increased hTERT expression in an endemic nigerian population
Source: Front Cell Infect Microbiol. 2026 Aug 6;16:1880313. doi: 10.3389/fcimb.2026.1880313 (PMC13490068; doi:10.3389/fcimb.2026.1880313)
Supplement: Supplementary file 1 [file DataSheet1.pdf]

Wakai et al.

Naturally Acquired Malaria Is Associated with Shorter Leukocyte Relative Telomere Length and Increased hTERT Expression in an Endemic Nigerian Population

SUPPLEMENTARY SHEET

Supplementary Table 1. *Primer sequences used for relative telomere length and hTERT expression analysis*

Target	Primer	Sequence (5′–3′)	Amplicon size
Telomere	tel1	GGTTTTTGAGGGTGAGGGTGAGGGTGAGGGTGAGGGT	~90–110 bp
Telomere	tel2	TCCCGACTATCCCTATCCCTATCCCTATCCCTATCCCTA	~90–110 bp
hTERT	hTERT-F	CCCATTTCATCAGCAAGTTTGG	95 bp
hTERT	hTERT-R	CTTGGCTTTCAGGATGGAGTAG	95 bp
GAPDH	GAPDH-F	GGTGTGAACCATGAGAAGTATGA	124 bp
GAPDH	GAPDH-R	GAGTCCTTCCACGATACCAAAG	124 bp
36B4 (RPLP0)	36B4u	CAGCAAGTGGGAAGGTGTAATCC	79 bp
36B4 (RPLP0)	36B4d	CCCATTCTATCATCAACGGGTACAA	79 bp

Supplementary Table 2. *Relative telomere length and hTERT expression by malaria status.*

Variable	Malaria-positive (n = 22)	Malaria-negative (n = 18)	p value
rTL (T/S ratio)	0.199 [0.172–0.216]	0.584 [0.554–0.636]	<0.001***
hTERT fold change	0.870 [0.770–1.050]	0.145 [0.133–0.170]	0.004**

Supplementary Table 3. *Relative telomere length, hTERT expression and selected haematological variables across parasite-density categories among malaria-positive participants.*

Variable	Low	Moderate	High	Hyperparasitaemia	p value
Relative telomere length (T/S ratio)	0.221 [0.203-0.395]	0.206 [0.204-0.210]	0.174 [0.120-0.191]	0.121 [0.069-0.183]	0.011*
hTERT fold change	0.830 [0.500-0.860]	0.900 [0.640-0.968]	0.890 [0.770-1.330]	1.370 [1.085-1.505]	0.426
Haemoglobin (g/dL)	11.49 [10.98-12.05]	12.02 [11.05-12.80]	8.96 [7.55-11.32]	9.13 [7.51-10.88]	0.168
Platelets (x10^9/L)	143.87 [95.02-167.08]	124.83 [100.23-146.43]	181.01 [142.00-186.35]	131.24 [108.18-151.56]	0.375

Supplementary Figure 1. qPCR Amplification, Melt Curve, and Melt Peak Profiles for Telomere, hTERT, 36B4 (RPLP0), and GAPDH Assays

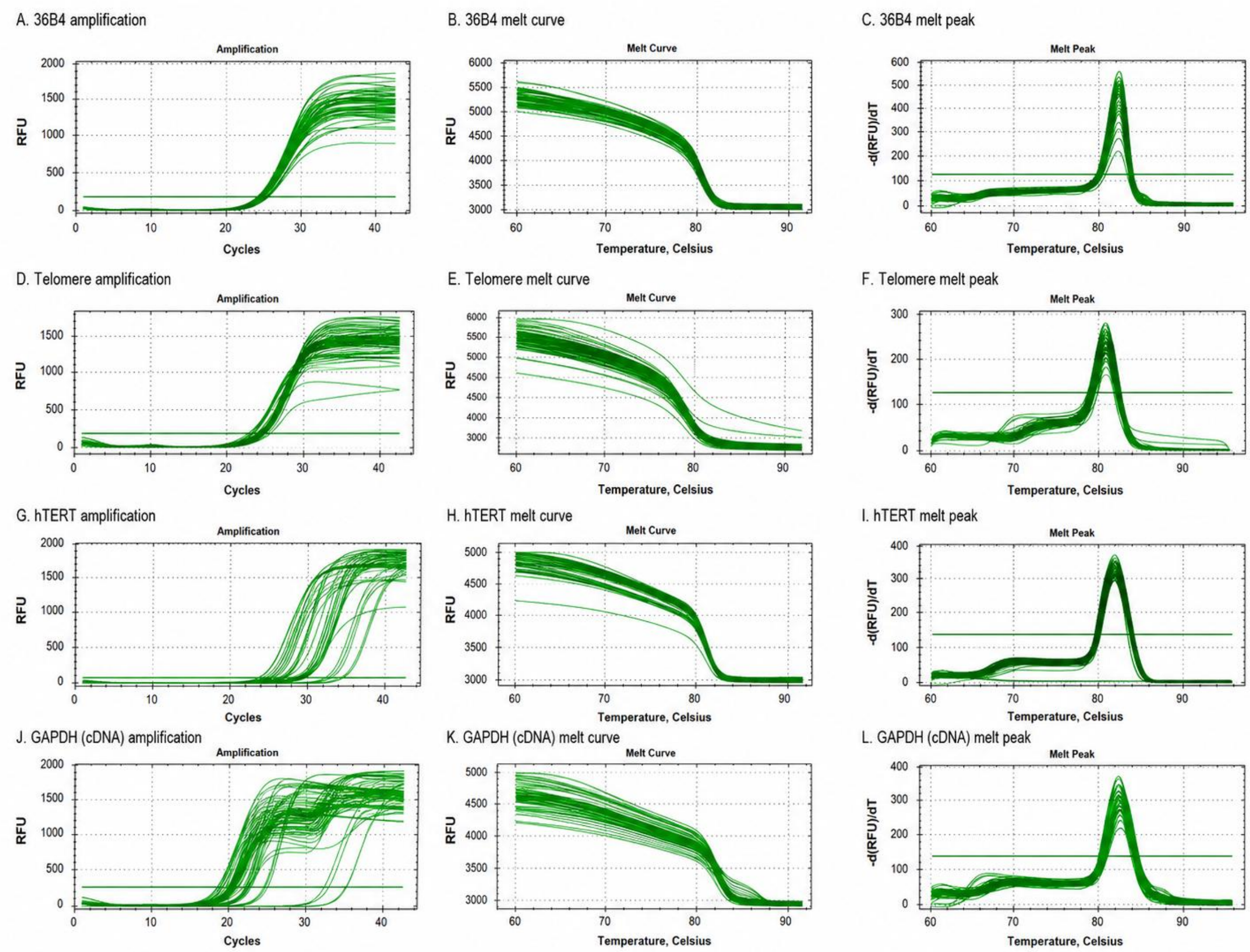

Supplementary Table 3. Intra-assay reproducibility of qPCR assays

Assay	Duplicate reactions (n)	Mean CV (%)	Range (%)
**Telomere**	28	0.46	0.00–2.11
**hTERT**	12	0.74	0.02–3.95
