# Supplementary material for: Naturally acquired malaria is associated with shorter leukocyte relative telomere length and increased hTERT expression in an endemic nigerian population
Source: Front Cell Infect Microbiol. 2026 Aug 6;16:1880313. doi: 10.3389/fcimb.2026.1880313 (PMC13490068; doi:10.3389/fcimb.2026.1880313)
Supplement: Supplementary file 2 [file Table1.docx]

**Wakai et al.**

**Naturally Acquired Malaria Is Associated with Shorter Leukocyte Relative Telomere Length and Increased hTERT Expression in an Endemic Nigerian Population**

**SUPPLEMENTARY SHEET**

***Supplementary Table 1.*** *Primer sequences used for relative telomere length and hTERT expression analysis*

| **Target** | **Primer** | **Sequence (5′–3′)** | **Amplicon size** |
| --- | --- | --- | --- |
| Telomere | tel1 | GGTTTTTGAGGGTGAGGGTGAGGGTGAGGGTGAGGGT | ~90–110 bp |
| Telomere | tel2 | TCCCGACTATCCCTATCCCTATCCCTATCCCTATCCCTA | ~90–110 bp |
| hTERT | hTERT-F | CCCATTTCATCAGCAAGTTTGG | 95 bp |
| hTERT | hTERT-R | CTTGGCTTTCAGGATGGAGTAG | 95 bp |
| GAPDH | GAPDH-F | GGTGTGAACCATGAGAAGTATGA | 124 bp |
| GAPDH | GAPDH-R | GAGTCCTTCCACGATACCAAAG | 124 bp |
| 36B4 (RPLP0) | 36B4u | CAGCAAGTGGGAAGGTGTAATCC | 79 bp |
| 36B4 (RPLP0) | 36B4d | CCCATTCTATCATCAACGGGTACAA | 79 bp |

***Supplementary Table 2.*** *Relative telomere length and hTERT expression by malaria status.*

| Variable | Malaria-positive (n = 22) | Malaria-negative (n = 18) | p value |
| --- | --- | --- | --- |
| rTL (T/S ratio) | 0.199 [0.172–0.216] | 0.584 [0.554–0.636] | <0.001*** |
| hTERT fold change | 0.870 [0.770–1.050] | 0.145 [0.133–0.170] | 0.004** |

*Supplementary Table 3. Relative telomere length, hTERT expression and selected haematological variables across parasite-density categories among malaria-positive participants.*

| **Variable** | **Low** | **Moderate** | **High** | **Hyperparasitaemia** | **p value** |
| --- | --- | --- | --- | --- | --- |
| Relative telomere length (T/S ratio) | 0.221 [0.203-0.395] | 0.206 [0.204-0.210] | 0.174 [0.120-0.191] | 0.121 [0.069-0.183] | 0.011* |
| hTERT fold change | 0.830 [0.500-0.860] | 0.900 [0.640-0.968] | 0.890 [0.770-1.330] | 1.370 [1.085-1.505] | 0.426 |
| Haemoglobin (g/dL) | 11.49 [10.98-12.05] | 12.02 [11.05-12.80] | 8.96 [7.55-11.32] | 9.13 [7.51-10.88] | 0.168 |
| Platelets (x10^9/L) | 143.87 [95.02-167.08] | 124.83 [100.23-146.43] | 181.01 [142.00-186.35] | 131.24 [108.18-151.56] | 0.375 |

***Supplementary Figure 1.*** qPCR Amplification, Melt Curve, and Melt Peak Profiles for Telomere, hTERT, 36B4 (RPLP0), and GAPDH Assays

**Supplementary Table 3.** Intra-assay reproducibility of qPCR assays

| Assay | Duplicate reactions (n) | Mean CV (%) | Range (%) |
| --- | --- | --- | --- |
| Telomere | 28 | 0.46 | 0.00–2.11 |
| hTERT | 12 | 0.74 | 0.02–3.95 |
